# Factors associated with late risks of breast cancer-specific mortality in the SEER registry

**DOI:** 10.1007/s10549-021-06233-4

**Published:** 2021-04-24

**Authors:** José P. Leone, Carlos T. Vallejo, Michael J. Hassett, Julieta Leone, Noah Graham, Nabihah Tayob, Rachel A. Freedman, Sara M. Tolaney, Bernardo A. Leone, Eric P. Winer, Nancy U. Lin

**Affiliations:** 1 Dana-Farber Cancer Institute, 450 Brookline Ave., Boston, MA 02215 USA; 2 Grupo Oncológico Cooperativo del Sur (GOCS), Neuquén, Argentina

**Keywords:** Hormone receptor, Estrogen receptor, Prognostic factors, Late, Recurrence, Relapse

## Abstract

**Purpose:**

Most reports describing the risk of late relapse in breast cancer (BC) have been based on selected patients enrolled into clinical trials. We examined population-based long-term risks of BC-specific mortality (BCSM), the risks of BCSM conditional on having survived 5 years, and factors associated with late BCSM.

**Methods:**

Using SEER, we identified women diagnosed with BC (T1-T2, N0-N2, M0) between 1990 and 2005 with known hormone receptor (HR) status. Kaplan–Meier analyses determined cumulative risks of BCSM. We performed Fine and Gray regression stratified by HR status.

**Results:**

We included 202,080 patients (median follow-up of 14.17 years). Of all BC deaths, the proportion that occurred after 5 years was 65% for HR-positive vs 28% for HR-negative (*p* < 0.001) BC. In HR-positive BC, the cumulative risks of BCSM during years 5–20 were 9.9%, 21.9%, and 38% for N0, N1, and N2 disease. For HR-negative BC, the risks were 7.9%, 12.2%, and 19.9%, respectively. For T1a/b, N0, HR-positive BC, the risk of BCSM was 6 times lower than the risk of non-BCSM. In N2, HR-positive BC, the risk of BCSM was 43% higher than the risk of non-BCSM. In adjusted Fine and Gray models stratified by HR status, the risks of BCSM conditional on having survived 5 years for both HR-positive and HR-negative depended on T-N status, age, and year of diagnosis. In HR-positive, the risks also depended on race and grade.

**Conclusion:**

The risks of BCSM beyond 5 years, although different, remain important for both HR-positive and HR-negative BC. Strategies to prevent early and late recurrences are warranted.

**Supplementary Information:**

The online version contains supplementary material available at 10.1007/s10549-021-06233-4.

## Introduction

Breast cancer is the most frequent malignancy and the leading cause of cancer-related death in women, representing approximately 25% of all malignancies and 15% of cancer-related deaths in the world [1, 2]. Although most patients are diagnosed with stage I-III disease, up to one-third will eventually develop distant recurrence [3]. The risk and timing of recurrence is influenced by patient and tumor characteristics as well as the receipt of appropriate local and systemic treatments [4–8]. Recurrences of breast cancer can occur both early (within 5 years of diagnosis) and late (more than 5 years from diagnosis). Quantifying the risk of early and late recurrences is a topic of major interest; hormone receptor (HR) status appears to be one of the strongest factors influencing risk. Early recurrences predominate in patients with HR-negative breast cancers, whereas late recurrences are common in patients with HR-positive tumors [9, 10]. Multiple tools are being evaluated to stratify the risk of early versus late recurrence, including risk calculators based on traditional clinicopathologic factors and gene expression-based assays [11–18].

However, most studies reporting on the risk of late recurrence in breast cancer have been based on selected patients enrolled into clinical trials, and population-based assessments of the risks of long-term breast cancer-specific mortality (BCSM) are lacking [9, 10, 19]. Further, studies evaluating this phenomenon have focused primarily on HR-positive breast cancer and less is known about longer term risk in HR-negative disease. The aims of this study were to report on population-based long-term risks of BCSM across HR-positive and HR-negative subtypes, and the risks of BCSM conditional on having survived 5 years. In addition, we aimed to identify factors associated with late deaths from breast cancer. Having more accurate estimates of the risks of late recurrence and long-term BCSM may inform clinical counseling in current breast cancer survivors and support the conceptual and statistical design of clinical trials focused on mitigating the risk of late recurrence.

## Patient and methods

### Data source and study design

We used data from the Surveillance, Epidemiology, and End Results (SEER) 18 registry (1973–2015) database [20]. We included women diagnosed with a first confirmed invasive breast cancer between 1990 and 2005. Because data on estrogen receptor (ER) and progesterone receptor (PR) status has been recorded since 1990, this was chosen as the initial year of diagnosis for inclusion. Given our aim of analyzing long-term risks of BCSM, we chose to include patients up until 2005, in order to allow for a minimum of 10 years of potential follow-up time.

To keep our cohort consistent with a recent meta-analysis [19], we included patients presenting with T1a, T1b, T1c, or T2 tumors; N0, N1, or N2; M0. Tumor size (T) and nodal status (N) were registered according to the American Joint Committee on Cancer staging system sixth edition. In addition, in order to most clearly differentiate HR status, we included only patients whose ER and PR status were coded as ‘positive’ or ‘negative’, and did not include cases coded as ‘borderline’ or ‘unknown’. We excluded patients who did not undergo surgery for their primary tumor (*n* = 1375), those with no or unknown regional nodal examination (*n* = 13,344), unknown number of positive lymph nodes (*n* = 549), or unknown cause of death (*n* = 1,576). In order to provide a more accurate reflection of outcomes attributable to the index cancer, we also excluded patients who had more than one primary malignancy over their lifetime (*n* = 106,474). Supplemental Figure 1 shows the inclusion and exclusion criteria for the patient population. The final sample size was 202,080 patients.

We evaluated the following variables as defined and categorized in Table 1: age at diagnosis, race, year of diagnosis, histology, tumor grade, tumor size, ER, PR, type of breast surgery, number of lymph nodes examined, number of positive lymph nodes, marital status, cause of death, and vital status. SEER reports four tumor grades, which we consolidated into three categories: grade I (well differentiated), grade II (moderately differentiated) and grade III/IV (poorly differentiated or anaplastic). Using the information from ER and PR status, we created a variable called hormone receptor (HR) which we categorized as positive (when either ER or PR was positive) or negative (when both ER and PR were negative).

**Table 1:** Patient characteristics

	Hormone receptor				Total	
	Positive		Negative			
	*N*	%	*N*	%	*N*	%
All patients	161,290	79.8	40,790	20.2	202,080	100.0
Age at diagnosis, y						
< 50	41,869	26.0	14,911	36.6	56,780	28.1
50–64	59,263	36.7	15,391	37.7	74,654	36.9
> 64	60,158	37.3	10,488	25.7	70,646	35.0
Race						
White	138,163	85.7	31,720	77.8	169,883	84.1
Black	10,224	6.3	5868	14.4	16,092	8.0
Other	12,459	7.7	3114	7.6	15,573	7.7
Unknown	444	.3	88	.2	532	.3
Grade						
I	33,953	21.1	1102	2.7	35,055	17.3
II	71,663	44.4	7402	18.1	79,065	39.1
III/IV	40,894	25.4	29,340	71.9	70,234	34.8
Unknown	14,780	9.2	2946	7.2	17,726	8.8
Histology						
Ductal	125,296	77.7	35,612	87.3	160,908	79.6
Lobular	14,725	9.1	677	1.7	15,402	7.6
Mixed ductal and lobular	14,997	9.3	954	2.3	15,951	7.9
Mucinous	23	.0	20	.0	43	.0
Papillary	411	.3	92	.2	503	.2
Carcinoma	5838	3.6	3435	8.4	9273	4.6
Stage						
I	87,761	54.4	16,706	41.0	104,467	51.7
II	62,362	38.7	20,499	50.3	82,861	41.0
III	11,167	6.9	3585	8.8	14,752	7.3
T						
T1a	8893	5.5	1746	4.3	10,639	5.3
T1b	33,940	21.0	4900	12.0	38,840	19.2
T1c	71,702	44.5	15,514	38.0	87,216	43.2
T2	46,755	29.0	18,630	45.7	65,385	32.4
N						
N0	110,108	68.3	27,014	66.2	137,122	67.9
N1	40,015	24.8	10,191	25.0	50,206	24.8
N2	11,167	6.9	3585	8.8	14,752	7.3
Surgery						
Partial mastectomy	91,565	56.8	21,716	53.2	113,281	56.1
Mastectomy	69,677	43.2	19,060	46.7	88,737	43.9
Unknown	48	.0	14	.0	62	.0
Marital status at diagnosis						
Single	17,801	11.0	4874	11.9	22,675	11.2
Married	96,717	60.0	25,005	61.3	121,722	60.2
Other	42,858	26.6	9868	24.2	52,726	26.1
Unknown	3914	2.4	1043	2.6	4957	2.5
Vital status						
Alive	105,361	65.3	25,805	63.3	131,166	64.9
Dead	55,929	34.7	14,985	36.7	70,914	35.1
Cause of death						
Alive	105,361	65.3	25,805	63.3	131,166	64.9
Breast cancer	21,054	13.1	8959	22.0	30,013	14.9
Other	34,875	21.6	6026	14.8	40,901	20.2
Breast cancer-related deaths						
Within 5 y	7315	34.7	6423	71.7	13,738	45.8
5–10 y	8334	39.6	1931	21.5	10,265	34.2
After 10 y	5405	25.7	605	6.8	6010	20.0

*y* years

Given that only deidentified data were used for this study, the study was considered exempt from Dana-Farber Cancer Institute’s Institutional Review Board review.

### Statistical analysis

For each variable, we excluded patients with unknown data from the comparative analysis. The primary endpoint for the study was BCSM, using SEER cause-specific death classification, and defined as the interval from initial breast cancer diagnosis to death from breast cancer or last follow-up for censored patients. Deaths due to causes other than breast cancer were considered as non-breast cancer-specific mortality (non-BCSM). We used Kaplan–Meier analyses to determine the effect of baseline variables as defined in Table 1 on cumulative risks of BCSM and non-BCSM, starting from year 5 after initial diagnosis. A Log-rank test was used to evaluate the difference between groups. We estimated the annual rate of events per 100 person-years. Fine and Gray regression was used to evaluate the association of multiple pre-specified variables with the risk of BCSM, when considering deaths from other causes as competing events [21]. Pre-specified variables included were T, N, age at diagnosis, race, tumor grade, HR status, and marital status. Given the long study period, we also included year of diagnosis in the multivariate models. We checked the proportional hazards assumption for each variable using Schoenfeld residual plots and identified that HR status violated the assumption whereas the other variables did not, therefore, the Fine and Gray model was stratified by HR status. All *P* values reported were two sided and *P* values < 0.05 were considered statistically significant. All statistical analyses were performed using STATA 12.0 (Stata Corporation, College Station, TX) and SPSS 20.0 (IBM Corporation, Armonk, NY).

## Results

### Patient characteristics

A total of 202,080 patients met inclusion/exclusion criteria for the final analytic cohort. Forty-five percent of cases (*n* = 91,505) were diagnosed between the years 1990 and 2000 and 55% (*n* = 110,575) were diagnosed between 2001 and 2005. Table 1 shows the distribution of patient characteristics according to nodal status. Overall, most patients were age d ≥ 50 years (71.9%, *n* = 145,300) and white race (84.1%, *n* = 169,883). The predominant tumor size was T1c (43.2%, *n* = 87,216) and 67.9% of patients (*n* = 137,122) were N0. Most patients (56.1%) underwent partial mastectomy. A total of 79.8% of patients (*n* = 161,290) had HR-positive tumors. Median age for HR-positive patients was 59 years (range, 17–102 years) and for HR-negative patients was 54 years (range, 18–99 years).

### Cumulative risk of BCSM

After a median follow-up of 14.17 years (IQR, 11.83 and 17.42 years), we observed 70,914 deaths of which 42.3% (*n* = 30,013) were due to breast cancer.

Among all breast cancer-related deaths, the proportion that occurred after 5 years from diagnosis was 65% for HR-positive breast cancer vs 28% for HR-negative breast cancer (*P* < 0.001). The distribution of breast cancer-related deaths over time is shown in Table 1. A total of 176,713 women were alive after 5 years from diagnosis and were included in the analysis of BCSM from year 5. Of these, 32,345 (18.3%) were HR-negative.

**Fig. 1:**
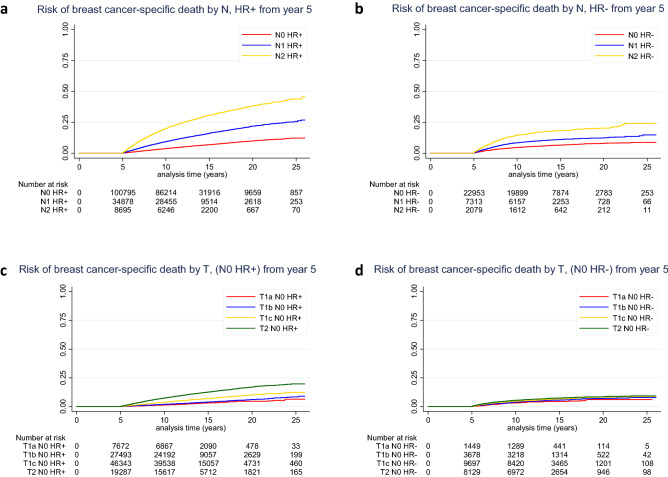
Unadjusted risk of breast cancer-specific death according to nodal status (among patients with **a** HR + breast cancer and **b** HR− breast cancer) and tumor size (among patients with **c** N0 HR + breast cancer and **d** N0 HR− breast cancer) starting from year 5 after diagnosis. *HR*− hormone receptor negative, *HR* + hormone receptor positive, *N* nodal status, *T* tumor size

The cumulative risk of BCSM from year 5 after initial breast cancer diagnosis according to N and HR status is shown in Fig. 1a, b and Table 2. The cumulative risk of BCSM from year 5 to year 20 ranged from 7.9% in HR-negative N0 breast cancer to 38% in HR-positive N2 breast cancer. For each nodal status group, the risk of BCSM at 20 years was higher for patients with HR-positive tumors compared with patients with HR-negative tumors (for N0: 9.9% v 7.9%, *P* = 0.17; for N1: 21.9% v 12.2%, *P* < 0.0001; for N2: 38% v 19.9%, *P* < 0.0001).

**Table 2:** Association of nodal status, tumor size, and grade with cumulative risk of breast cancer-specific mortality and non-breast cancer-specific mortality from years 5 to 20

	BCSM	Non-BCSM
	Cumulative risk (%)	Cumulative risk (%)
	y 5–20	y 5–20
Nodal status		
HR +		
N0	9.9	32.2
N1	21.9	26.5
N2	38	26.5
HR−		
N0	7.9	23.2
N1	12.2	19.7
N2	19.9	22.6
Tumor size among N0 only		
HR +		
T1a	4.6	29.5
T1b	5.9	32.9
T1c	10.1	31.6
T2	16.8	33.8
HR−		
T1a	6.1	24.3
T1b	6.8	26.6
T1c	8.1	22.8
T2	8.4	21.9
Tumor grade among N0 only		
HR +		
Grade I	5.7	32.6
Grade II	9.9	32.3
Grade III/IV	13.5	29.2
HR−		
Grade I	6.6	32.2
Grade II	10.8	29.6
Grade III/IV	6.9	19.8

*BCSM* breast cancer-specific mortality, *HR*− hormone receptor negative, *HR* + hormone receptor positive, *y* years

The impact of tumor size by HR status on cumulative risk of BCSM among patients with N0 disease is shown in Fig. 1c, d and Table 2. The cumulative risks of BCSM from year 5 to year 20 for T1a and T1b HR-positive tumors were 4.6% and 5.9%, respectively, and 10.1% and 16.8% for T1c and T2 HR-positive tumors. In HR-negative disease, the cumulative risk of BCSM during the same period of time ranged from 6.1 to 8.4%. The analyses of tumor grade by HR status among patients with N0 disease are shown in Supplemental Figure 2a, b and Table 2. The cumulative risks of BCSM from year 5 to 20 among HR-positive disease with grade I, grade II, and grade III/IV were as follows: 5.7%, 9.9%, and 13.5%, respectively. Among patients with HR-negative disease, the cumulative risk of BCSM during that time ranged from 6.6 to 10.8%.

Table 3 shows the multivariate Fine and Gray analyses stratified by HR status. Among patients with HR-positive breast cancer, independent prognostic factors for BCSM conditional on having survived 5 years included tumor size, nodal status, age at diagnosis, race, tumor grade, marital status, and year of diagnosis. In contrast, among patients with HR-negative breast cancer, independent prognostic factors for BCSM conditional on having survived 5 years included tumor size, nodal status, age at diagnosis, and year of diagnosis, but race was not significantly associated with breast cancer survival outcomes.

**Table 3:** Multivariate analysis for breast cancer-specific mortality starting from year 5 after diagnosis stratified by hormone receptor status

Variable	Fine and Gray Model for HR +			Fine and Gray Model for HR−		
	*P*	Hazard ratio	95.0%CI for hazard ratio	*P*	Hazard ratio	95.0%CI for hazard ratio
T						
T1a		Reference			Reference	
T1b	0.013	1.204	1.040–1.392	0.759	1.045	0.789–1.385
T1c	< 0.001	1.932	1.683–2.218	0.007	1.424	1.100–1.842
T2	< 0.001	3.052	2.655–3.508	< 0.001	1.826	1.410–2.363
N						
N0		Reference			Reference	
N1	< 0.001	1.949	1.869–2.032	< 0.001	1.721	1.562–1.896
N2	< 0.001	3.390	3.210–3.580	< 0.001	2.742	2.411–3.117
Age at diagnosis						
< 50 years		Reference			Reference	
50–64 years	0.007	0.940	0.899–0.983	0.006	1.152	1.042–1.273
> 64 years	0.002	1.077	1.027–1.130	< 0.001	1.397	1.244–1.568
Race						
White		Reference			Reference	
Black	< 0.001	1.290	1.206–1.379	0.072	1.123	0.990–1.273
Other	0.229	0.960	0.897–1.026	0.181	0.896	0.762–1.053
Grade						
Grade I		Reference			Reference	
Grade II	< 0.001	1.718	1.679–1.828	0.004	1.453	1.124–1.879
Grade III/IV	< 0.001	2.125	1.991–2.268	0.495	0.915	0.710–1.180
Marital status						
Single		Reference			Reference	
Married	< 0.001	0.887	0.838–0.939	0.383	0.941	0.821–1.079
Other	0.738	0.989	0.928–1.054	0.786	1.021	0.878–1.188
Year of diagnosis						
Each additional year	< 0.001	0.944	0.940–0.948	< 0.001	0.946	0.936–0.955

*CI* confidence interval, *HR* + hormone receptor positive, *HR*− hormone receptor negative

### Annual rates of BCSM

**Fig. 2:**
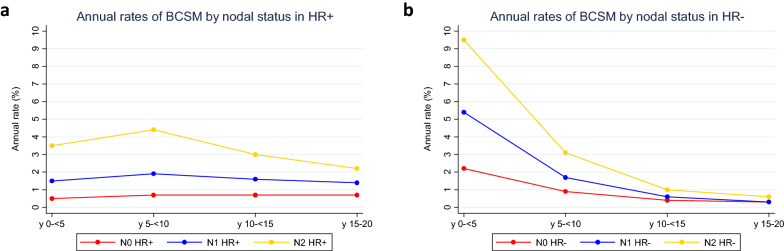
Annual rate of breast cancer-specific mortality according to nodal status among patients with **a** HR + breast cancer and **b** HR− breast cancer from the time of initial diagnosis. *HR*− hormone receptor negative, *HR* + hormone receptor positive, *y* years

We evaluated annual rates of BCSM for the period of time from initial diagnosis to 20 years after diagnosis, divided into 5 year intervals. The results for each nodal status category by HR status are shown in Fig. 2a, b and Table 4. Among patients with N0 HR-positive breast cancer, we observed a constant rate of annual events throughout the years 0 to 20. Patients with N1 and N2 HR-positive breast cancer reached a maximum rate of events at year 5–10, followed by a decrease up to year 20. In contrast, for each nodal status category within HR-negative breast cancer, annual rates of BCSM peaked within the first 5 years from diagnosis, followed by a sharp decline up to year 20. Overall, the annual rates of BCSM from year 5 to 20 were lower for patients with HR-negative breast cancer compared with those for HR-positive breast cancer.

**Table 4:** Annual rate of breast cancer-specific mortality by HR and nodal status from years 0 to 20, divided in 5 year intervals

	Annual rate (%)			
	y 0– < 5	y 5– < 10	y 10– < 15	y 15–20
HR +				
N0	0.5	0.7	0.7	0.7
N1	1.5	1.9	1.6	1.4
N2	3.5	4.4	3	2.2
HR−				
N0	2.2	0.9	0.4	0.3
N1	5.4	1.7	0.6	0.3
N2	9.5	3.1	1	0.6

*HR* + hormone receptor positive, *HR*− hormone receptor negative, *y* years

### Cumulative risk of non-BCSM

We evaluated non-BCSM for various subgroups of patients and estimated cumulative risk from year 5 to 20 (Table 2, Supplemental Figure 3a-c). Compared with the risk of BCSM from years 5–20, the risk of non-BCSM during the same period was approximately 3 times higher in both N0 HR-positive and N0 HR-negative breast cancer. The risk of non-BCSM was 1.2 and 1.6 times higher than the risk of BCSM in patients with N1 HR-positive and N1 HR-negative disease, respectively. Patients with N2 HR-negative breast cancer had similar risk of BCSM and non-BCSM from years 5–20, whereas patients with N2 HR-positive disease had a relative 30% lower risk of non-BCSM than BCSM.

## Discussion

Deaths beyond 5 years from initial breast cancer diagnosis remain a significant clinical challenge. Our study was designed to evaluate population-based long-term risks of BCSM for both HR-positive and HR-negative breast cancers. In addition, we report the risk of non-BCSM, the risk of BCSM conditional on having survived 5 years, and the factors associated with late breast cancer deaths.

In HR-positive breast cancer, the risk of BCSM was constant for node-negative patients with an annual rate of events around 0.7%. The risk was significantly higher in node-positive patients, in whom the annual rate of events was not constant over time. Patients with N1 and particularly N2 disease experienced a higher rate of BCSM within the first 10 years from diagnosis and consequently a lower rate after 10 years. However, despite this lower risk beyond 10 years, node-positive patients still remained at a higher risk of BCSM even after 10 years from diagnosis when compared with their node-negative counterparts, with annual rates of events that were twice higher for N1 and three times higher for N2 than in node-negative patients. The population-based source of these data provides robust and clinically valuable estimates of BCSM in various subset of patients with HR-positive breast cancer. A recent meta-analysis of 62,923 women with HR-positive breast cancer, who were disease-free after 5 years of planned endocrine therapy, reported that the risks of distant recurrence from 5 to 20 years were 15% for N0, 23% for N1, and 38% for N2 [19]. In our study, the risks of BCSM from 5 to 20 years were 9.9%, 21.9%, and 38% for N0, N1, and N2, respectively. Similar results were reported in two prior studies, although with smaller sample sizes and shorter follow-up [22, 23]. Our study showed that most breast cancer deaths in HR-positive breast cancer occur after 5 years from initial diagnosis (65%), whereas only 28% of breast cancer deaths occur during the same time period in patients with HR-negative disease. This is likely due to the longer survival post-distant recurrence of HR-positive breast cancer compared with HR-negative disease [22].

In HR-negative breast cancer, the risk of BCSM was highest within the first 5 years from diagnosis, regardless of nodal status. However, beyond 5 years, the risk of BCSM remained important. For example, for patients with N2 disease, the absolute risk of BCSM between years 5 and 20 was 19.9%. There is a paucity of data on long-term outcomes for patients with HR-negative disease, and our study provides essential risk estimates for this subset of breast cancer. A pooled analysis that included 1148 ER-negative breast cancers showed that the annual hazard of recurrence was higher for ER-negative disease than for ER-positive disease within the first 5 years from diagnosis, and that beyond 5 years, patients with ER-positive breast cancer had higher annual hazard of recurrence than those with ER-negative disease [9]. A retrospective analysis of 873 patients with triple-negative breast cancer who remained disease-free at 5 years from diagnosis had a 15 year relapse-free interval of 95% [24].

The analysis of non-BCSM from our study helps to integrate the risks of BCSM into the perspective of the overall risk of death for various groups of patients. In patients with lower-risk tumors such as T1a or T1b, N0, HR-positive breast cancer, the risk of BCSM from 5 to 20 years was approximately 6 times lower than the risk of non-BCSM. Patients with N1, HR-positive breast cancer had similar risks of BCSM and non-BCSM from 5 to 20 years. In contrast, in patients with higher-risk tumors such as N2, HR-positive breast cancer, the risk of BCSM from years 5 to 20 was 43% higher than the risk of non-BCSM. These results could assist in the refinement of guidelines for treatment and follow-up of breast cancer patients that take into account both breast cancer risk and competing risks for mortality.

The multivariate Fine and Gray analyses stratified by HR status aimed to identify specific factors associated with BCSM conditional on having survived 5 years, while accounting for competing risks of death. Tumor size and nodal status were independently and strongly associated with BCSM both in HR-positive and in HR-negative breast cancer. Patients age 65 years and older had worse BCSM regardless of HR status, a finding that is consistent with results from a recent study [25]. The multivariate analysis also showed a significant improvement in BCSM over time, with a hazard ratio of 0.94 per year in both HR-positive and HR-negative breast cancer. This may be due to improvements in screening and early breast cancer detection, and advances in systemic therapy over the study period. Overall, our results underscore the role of traditional clinicopathologic factors, which remain strongly associated with BCSM even beyond 5 years from breast cancer diagnosis. While this was accepted in HR-positive disease, our study confirms that tumor size and nodal status also influence BCSM beyond 5 years in HR-negative breast cancer.

Our study has a number of important strengths. To our knowledge, this is the largest population-based analysis conducted to date on the risks of long-term BCSM with estimates at 20 years from initial diagnosis. The inclusion of HR-negative breast cancer allows us to provide risk estimates for that subgroup which has been understudied. The analysis of factors associated with BCSM conditional on having survived 5 years provides a unique opportunity to analyze these associations in clinically relevant subgroups at risk of late events. Finally, the population-based design of our study confers strong external validity to our results.

We also acknowledge a number of study limitations. First, we do not have data on the use of systemic therapies or adherence to treatment, which substantially impact outcomes. However, our data are consistent with the results for women with HR-positive breast cancer in a meta-analysis of patients treated in clinical trials [19]. Another limitation is the lack of information about HER2 status. Although adjuvant anti-HER2 therapies did not become standard until 2005, trastuzumab became standard therapy for metastatic HER2-positive breast cancer around 1999. Thus, some of the late BCSM we observed in HR-positive and in particular in HR-negative cases may be due to HER2-positive disease treated with trastuzumab with an anticipated longer survival than those with triple-negative disease. The long-term follow-up of the pivotal adjuvant trastuzumab trials showed recurrences in HER2-positive cases between years 5 and 10, and rates of late recurrence in HER2-positive disease are higher in HR-positive/HER2-positive subtype [26].

Further, SEER does not collect information on cancer recurrences, which would have provided additional risk estimates if available. The risks reported in our study may not accurately reflect the risks of patients diagnosed with breast cancer in more recent years, as the prognosis of breast cancer has improved in recent years with earlier diagnosis and better treatment modalities.

In conclusion, we observed that for HR-positive breast cancer, risks of BCSM remain high beyond 5 years from diagnosis and depend on tumor size, nodal status, age, race, and tumor grade. For HR-negative breast cancer, risks of BCSM are highest within 5 years from diagnosis; however, risks beyond 5 years are still important and depend on tumor size, nodal status, and age. Our results provide essential estimates of late BCSM events that could be used in the statistical design of clinical trials evaluating late endpoints, both in HR-positive and HR-negative breast cancer. We identified a group of lower-risk patients in whom the risk of BCSM was 6 times lower than the risk of non-BCSM, and a group of higher-risk patients in whom the risk of BCSM was higher than the risk of non-BCSM. Our results underscore the need for better therapies and strategies to prevent early and late recurrences in both HR-positive and HR-negative breast cancer.

## Supplementary Information

Below is the link to the electronic supplementary material.

Supplementary file1 (DOCX 119 kb)
