# Correction to: Factors associated with late risks of breast cancer‑specific mortality in the SEER registry

**DOI:** 10.1007/s10549-021-06268-7

**Published:** 2021-07-06

**Authors:** José P. Leone, Carlos T. Vallejo, Michael J. Hassett, Julieta Leone, Noah Graham, Nabihah Tayob, Rachel A. Freedman, Sara M. Tolaney, Bernardo A. Leone, Eric P. Winer, Nancy U. Lin

**Affiliations:** 1 Dana-Farber Cancer Institute, 450 Brookline Ave., Boston, MA 02215 USA; 2 Grupo Oncológico Cooperativo del Sur (GOCS), Neuquén, Argentina

## Correction to: Breast Cancer Research and Treatment https://doi.org/10.1007/s10549-021-06233-4

The article “Factors associated with late risks of breast cancer‑specific mortality in the SEER registry”, written by José P. Leone, Carlos T. Vallejo, Michael J. Hassett, Julieta Leone, Noah Graham, Nabihah Tayob, Rachel A. Freedman, Sara M. Tolaney, Bernardo A. Leone, Eric P. Winer and Nancy U. Lin, was originally published electronically on the publisher’s internet portal on April 24, 2021 without open access. With the author(s)’ decision to opt for Open Choice the copyright of the article changed on May 11, 2021 to © The Author(s) 2021 and the article is forthwith distributed under a Creative Commons Attribution 4.0 International License, which permits use, sharing, adaptation, distribution and reproduction in any medium or format, as long as you give appropriate credit to the original author(s) and the source, provide a link to the Creative Commons licence, and indicate if changes were made. The images or other third party material in this article are included in the article’s Creative Commons licence, unless indicated otherwise in a credit line to the material. If material is not included in the article’s Creative Commons licence and your intended use is not permitted by statutory regulation or exceeds the permitted use, you will need to obtain permission directly from the copyright holder. To view a copy of this licence, visit http://creativecommons.org/licenses/by/4.0.

The original article has been corrected.

