# Supplementary material for: Factors associated with late risks of breast cancer-specific mortality in the SEER registry
Source: Breast Cancer Res Treat. 2021 Apr 24;189(1):203–12. doi: 10.1007/s10549-021-06233-4 (PMC8302525; doi:10.1007/s10549-021-06233-4)
Supplement: Supplementary file 1 — Supplementary file1 (DOCX 119 kb) [file 10549_2021_6233_MOESM1_ESM.docx]

**SUPPLEMENT**

**Factors associated with twenty-year risks of breast cancer-specific mortality in the SEER Registry**

Supplemental Figures: 3

**Supplemental Figure 1:** Flow diagram of patient population


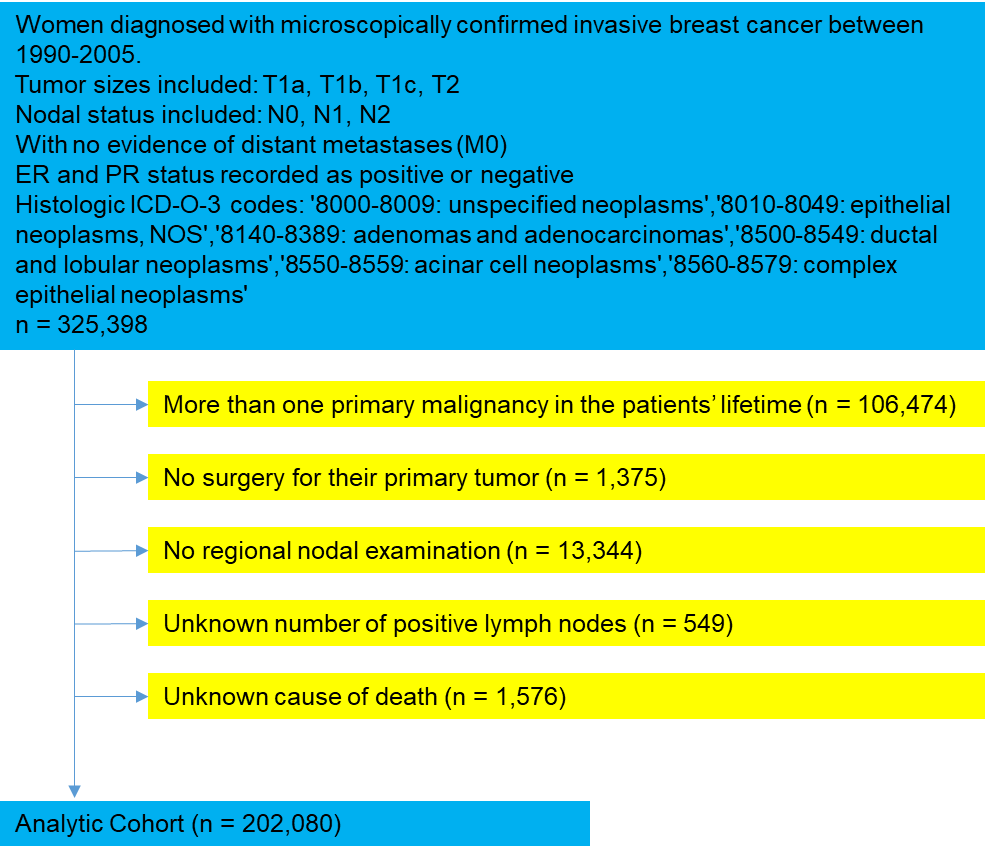
Abbreviations: ICD-O-3, international classification of diseases for oncology – 3^rd^ edition; NOS, not otherwise specified.

**Supplemental Figure 2:** Unadjusted risk of breast cancer-specific death according to tumor grade among patients with a) N0 HR-positive breast cancer and b) N0 HR-negative breast cancer starting from year 5 after diagnosis. HR, hormone receptor; y, years

**a.**

**b.**

**Supplemental Figure 3:** Unadjusted risk of non-breast cancer-specific death by a) nodal status and by HR status, b) tumor size and HR status among patients with N0 breast cancer, and c) tumor grade and HR status among patients with N0 breast cancer starting from year 5 after diagnosis. HR, hormone receptor; non-BCSM, non-breast cancer specific mortality; y, years

**a.**

**c.**

**b.**
